# Wear and corrosion resistance of Co–P coatings: the effects of current modes

**DOI:** 10.1039/c7ra10830c

**Published:** 2018-01-03

**Authors:** Ruiqian Li, Yuanyuan Hou, Qiujing Dong, Peibo Su, Pengfei Ju, Jun Liang

**Affiliations:** School of Chemistry and Materials Engineering, Fuyang Normal University Fuyang Anhui 236037 China; State Key Laboratory of Solid Lubrication, Lanzhou Institute of Chemical Physics, Chinese Academy of Sciences Lanzhou 730000 China jliang@licp.cas.cn; Shanghai Aerospace Equipment Manufacturer Shanghai 200245 China

## Abstract

In this work, Co–P coatings were deposited from a chloride-based bath by direct current (DC), pulse current (PC) and pulse reverse current (PRC) methods, respectively. The effects of current modes on the microstructure, composition, microhardness, wear resistance and corrosion resistance of the Co–P coatings were explored. Results showed that the P content in the Co–P coatings increased and the surface roughness decreased in the sequence of DC, PC and PRC methods. The coatings with low P content deposited by DC and PC methods are crystalline with fcc and hcp structures, respectively, while the coating with high P content deposited by the PRC method is amorphous. Comparing to DC and PC methods, the PRC method can evidently improve the microhardness, wear resistance and corrosion resistance of Co–P coatings. The excellent wear and corrosion resistance of the Co–P coatings deposited by the PRC method could be attributed to its high P content, smooth surface and amorphous structure.

## Abstract 2

The P content in the Co–P coatings increased in the sequence of DC, PC and PRC methods. The PRC Co–P coating has better wear and corrosion resistance than DC and PC Co–P coatings.

## Graphical abstract

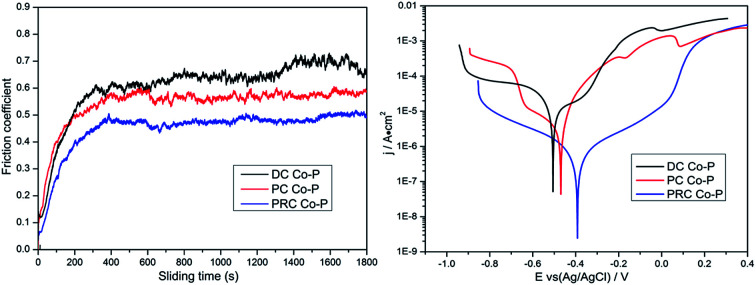

## 1. Introduction

Co–P coatings have been identified as suitable materials for replacement of environmentally-unfriendly hard chromium due to their excellent wear resistance, corrosion resistance and high thermal stability.^1–5^ The properties of Co–P coatings are mainly influenced by the P content and phase structures of alloy coatings. It was reported that Co–P coatings with high P content and amorphous structure exhibit excellent corrosion resistance.^6,7^

There are many factors that affect the P content and phase structures of Co–P coatings, such as electrical parameters (current modes and current density), plating bath composition (P source concentration, additive type and pH value) and bath temperature. Among these variables, current mode is a flexible and efficient variable to modify the P content and phase structures of alloy coatings. Ezhilselvi *et al.*^1,7^ demonstrated that the Co–P coatings deposited by PC method have better corrosion resistance than those by DC method. Pulse current (PC) and pulse reverse current (PRC) are the two most common types of pulsed method. In spite of the deposition of Co–P by DC and PC methods has been extensively studied,^1–3,7^ however, to the best of our knowledge, rarely attention has been paid to the deposition of Co–P alloy coating by PRC method. It was reported that alloy coatings deposited by PRC method exhibits higher corrosion resistance and wear resistance than that deposited by PC methods.^8–12^ The PRC mode contains two electrode reaction processes in a period, one is a cathode process, which is similar to that of PC mode; the other is an anode process, which not only inhibited the growth of grains, but also dissolved the unsteady phases. In the process of electrodeposition, a regular cathode and anode process are a best way to modify the composition and microstructures, and then improve the properties of alloy coatings. Generally, the Co–P coatings should satisfy both the wear resistance and corrosion resistance in the practical conditions. However, it's a great pity that the corrosion and wear resistance of Co–P coatings were rarely researched simultaneously.

In this work, Co–P coatings were electrodeposited by DC, PC and PRC methods on a copper substrate from chloride bath. The effect of current modes (DC, PC and PRC) on the microstructure, composition, corrosion and wear resistance of the Co–P coatings was investigated in detail.

## 2. Experimental

### 2.1 Electrodeposition process

**Fig. 1:**
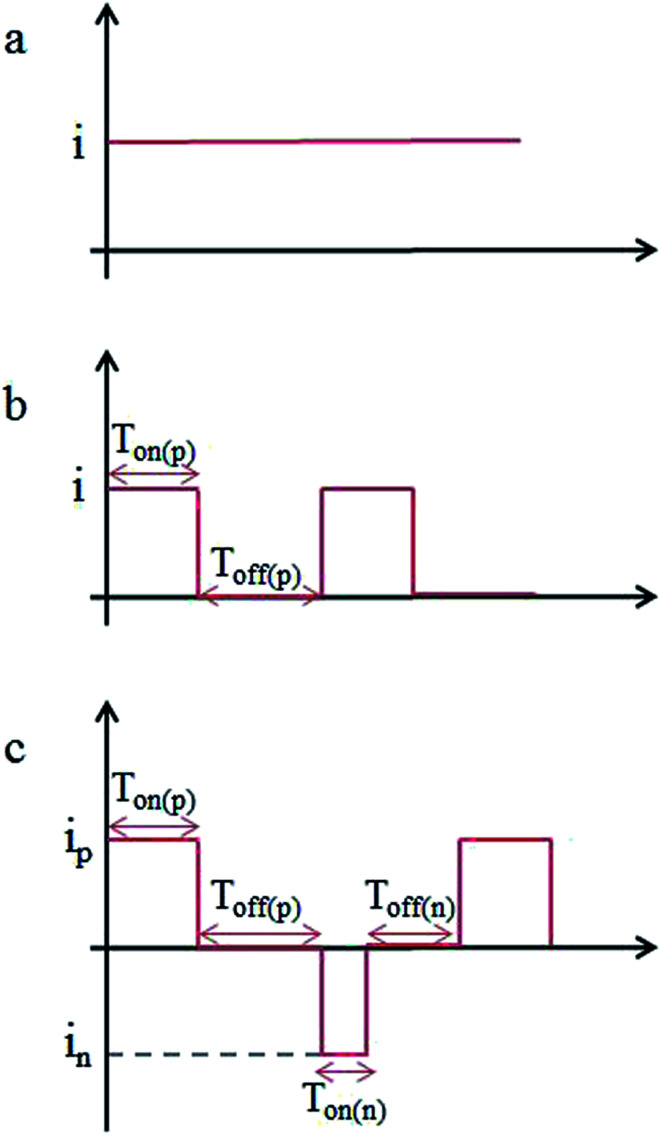
Schematic diagram of: (a) DC, (b) PC and (c) PRC.

Co–P coatings were electrodeposited from a chloride-based bath. The following bath was used: cobalt chloride, 25 g L^−1^; sodium hypophosphite, 4 g L^−1^. The bath temperature was adjusted in the range of 50 ± 2 °C using oil bath. The pH of the bath was kept constant at 2.0 ± 0.1 by the addition of 10 wt% HCl. Cu plate with surface area of 7 × 30 mm was used as cathode, whilst a pure Co plate was used as anode. The Cu plates were polished with 800#, 1200# and 1500# sandpaper, cleaned in acetone and activated in 5 wt% HCl for 30 s. The Co–P coatings were deposited by DC, PC and PRC methods for 2 h, respectively. The schematic view of DC, PC and PRC methods are presented in Fig. 1, representing operating parameters such as the forward and reverse peak current density (*i*_p_, *i*_n_), the forward and reverse pulse on-time (*T*_on(p)_, *T*_on(n)_) and the forward and reverse pulse off-time (*T*_off(p)_, *T*_off(n)_). The detailed parameters of DC, PC and PRC methods are listed in Table 1.

**Table 1:** Process parameters for electrodeposition of Co–P coatings

Current types	Electrodeposition parameters
**Direct current (DC) electrodeposition**	
Current density	15 mA cm^−2^

**Pulse current (PC) electrodeposition**	
Peak current density	15 mA cm^−2^
Pulse on-time	400 μs
Pulse off-time	1600 μs

**Pulse reverse current (PRC) electrodeposition**	
Peak current density of forward pulse	15 mA cm^−2^
Peak current density of reverse pulse	15 mA cm^−2^
Forward pulse on-time	400 μs
Forward pulse off-time	1600 μs
Reverse pulse on-time	100 μs
Reverse pulse off-time	1900 μs

### 2.2 Coating characterization

The crystal structure of the Co–P coatings was determined by using X-ray diffraction (XRD, D/MAX-2400, Japan) with a Cu target (*λ* = 0.15406 nm). The surface morphology and composition of Co–P coatings were characterized by scanning electron microscopy (SEM, JEOL, JSM-5600LV) with X-ray energy dispersive spectroscopy (EDS, Kevex). The surface morphology and roughness of Co–P coatings were determined by atomic force microscope (AFM, Solver PRO-M, NT-MDT).

Vickers microhardness test was carried out by a microhardness tester (HXD-1000B) at a load of 50 N for 5 s. Friction and wear tests sliding against an AS14 steel ball (*Ø* 6 mm) were performed with a ball-on-plate type wear tester (UMT, Tribolab) at room temperature with dry sliding conditions. The tests were performed at a constant applied load of 2 N and a sliding distance of 10 mm with a sliding frequency of 5 Hz for 30 min.

The corrosion behavior of the Co–P coatings were studied by potentiodynamic polarization (Tafel) and electrochemical impedance spectroscopy (EIS) on an Autolab PGSTAT302N electrochemical workstation in 3.5 wt% NaCl solution. An Ag/AgCl (saturated with KCl) reference electrode (SCE) and a Pt plate as counter-electrode were used in the tests. The impedance measurements were performed at open circuit potential (OCP) with an AC amplitude of 10 mV (peak to peak) in the frequency range of 30 kHz to 10 mHz. The specimens were immersed in the 3.5 wt% NaCl solution for about 60 min before Tafel and EIS tests to ascertain stable open circuit potentials. All the electrochemical measurements were repeated at least three times until good reproducibility of the data were obtained. All the electrochemical tests were carried out at room temperature (20 ± 2 °C).

## 3. Results and discussion

### 3.1 Compositions and microstructure

The composition of Co–P coatings were examined by EDS and the results are shown in Table 2. It is clearly seen that the P content of Co–P coatings deposited by DC, PC, and PRC methods (denoted as “DC Co–P”, “PC Co–P” and “PRC Co–P” coating in the following context, respectively) are 1.3 wt%, 3.4 wt% and 12.0 wt%, respectively. The higher P content in the coatings deposited by the PC and PRC methods can be attributed to the hypophosphite anions diffuse to the cathode during the off-time, resulting in the enrichment of hypophosphite anions.

**Table 2:** Composition of Co–P coatings examined by EDS

Element	DC Co–P	PC Co–P	PRC Co–P
Co (wt%)	98.7	96.6	88.0
P (wt%)	1.3	3.4	12.0

**Fig. 2:**
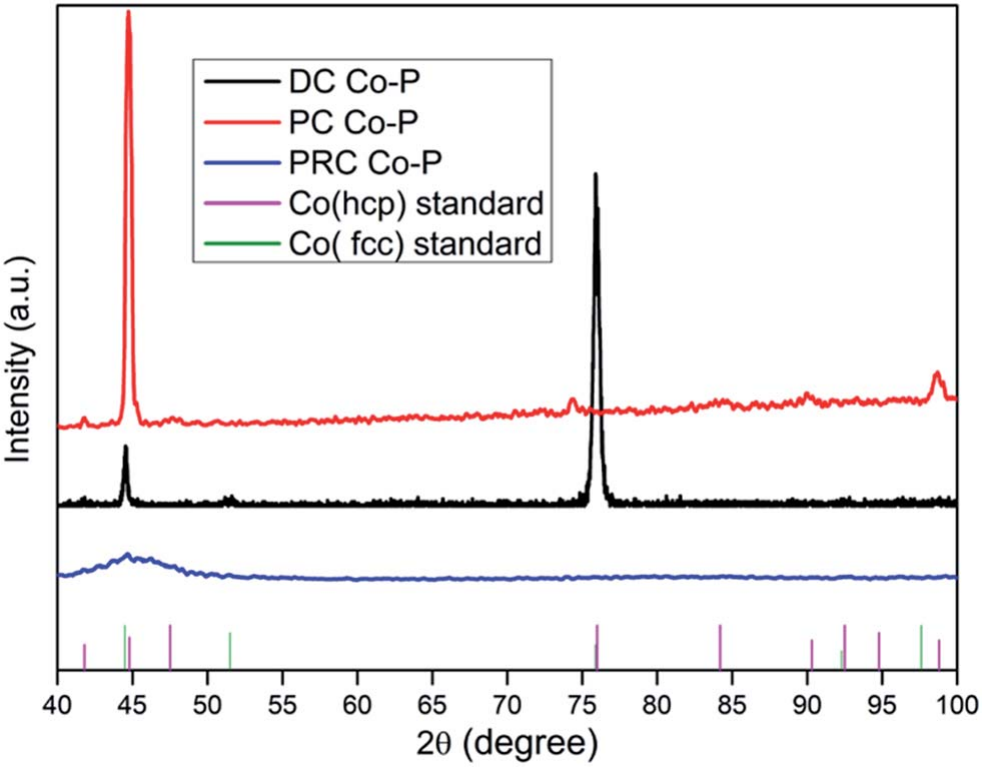
The XRD spectra of Co–P coatings deposited by DC, PC and PRC methods.

The XRD patterns for the DC Co–P, PC Co–P and PRC Co–P coatings are presented in Fig. 2. It can be seen that there are three diffraction peaks at 44.6°, 51.5° and 75.7° assigned to the (111), (200) and (110) planes of Co for the DC Co–P coating. These peaks are in good agreement with those of the reference patterns for a mixed fcc/hcp phase with higher ratio of fcc phase structure. The highest intensity peak at 2*θ* of 75.7° indicates that the DC Co–P coating has a preferred orientation of (110) plane. The XRD pattern of PC Co–P coating show diffraction peaks at 41.7°, 44.8°, 47.5°, 90.3° and 98.8° corresponding to (100), (002), (101), (200) and (004) planes of hexagonal close packed (hcp) Co. It is evident that the PC Co–P coating is crystalline with the preferred orientation of (002) plane. The PRC Co–P coating shows an amorphous structure due to the higher phosphorous content.^7^ The average crystallite size of DC Co–P coating and PC Co–P coating evaluated from Debye–Scherrer equation are 24.6 and 15.7 nm, respectively. The refinement of grains could be attributed to the improvement of current distribution and mass transfer under PC condition. Based on the Tafel equation, a larger current density corresponds to a higher overpotential, which decreases the activation energy of nucleation, and causes an increased nucleation rate.^13^

**Fig. 3:**
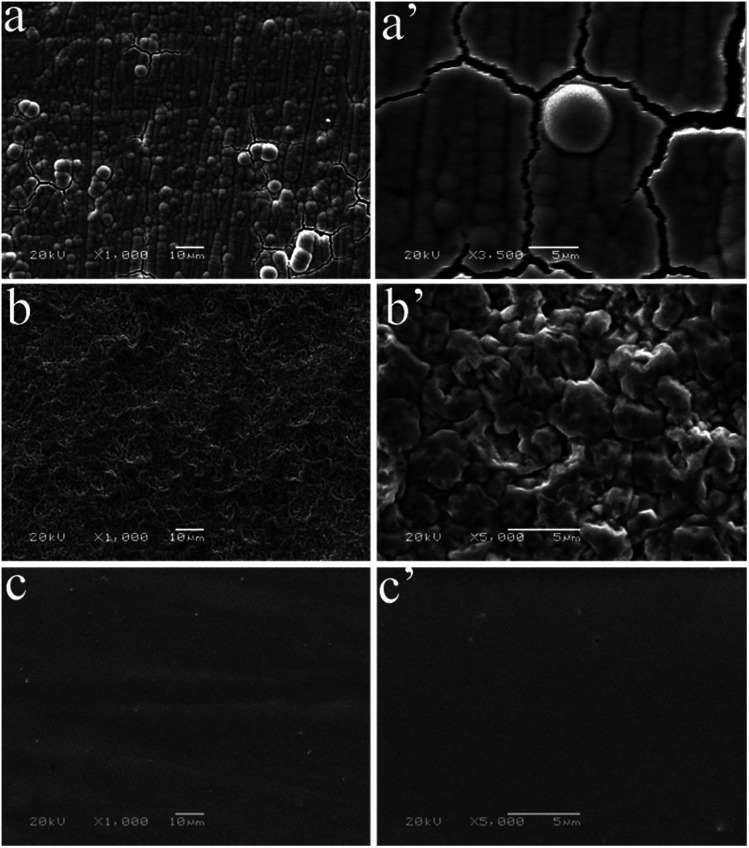
SEM images of surface morphology of Co–P coatings deposited by (a) DC, (b) PC and (c) PRC methods.

The surface morphologies of Co–P coatings deposited by DC, PC and PRC methods are shown in Fig. 3a–c, respectively. It is clearly observed that the DC Co–P coating shows a spherical cluster structure with a size in the range of a few micrometers and some cracks and voids can be seen on the coating surface (Fig. 3a and a′). For the PC Co–P coating, the surface is characterized by irregular polyhedron structure and there are some small voids (Fig. 3b and b′). The Co–P coating deposited by PRC reveals a smooth and compact surface without any cracks and voids.

**Fig. 4:**
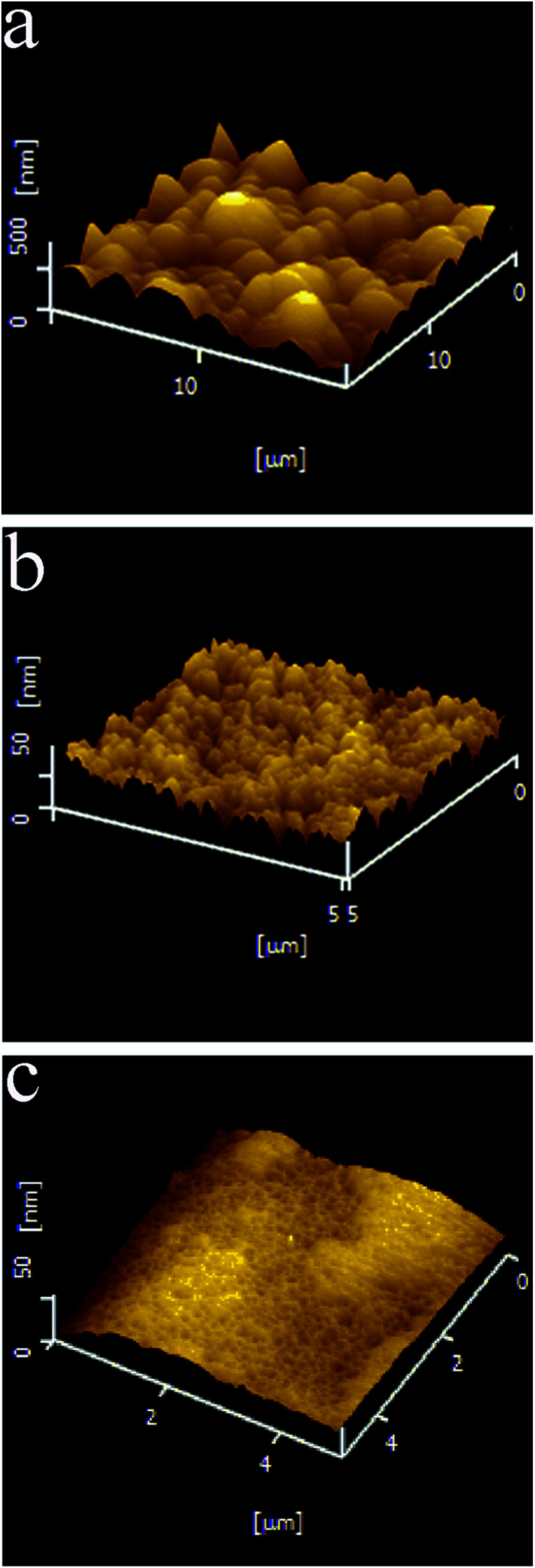
AFM images of Co–P coatings deposited by (a) DC, (b) PC and (c) PRC methods.

**Fig. 5:**
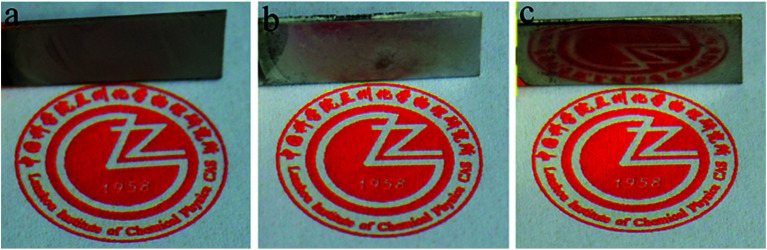
The photos of Co–P coatings deposited by (a) DC, (b) PC and (c) PRC methods.

The surface characterizations of the three types of Co–P coatings are further investigated by AFM. As shown in Fig. 4a, spherical protrusions are clearly visible on the surfaces of the DC Co–P coating with an average surface roughness (*R*_a_) of about 97.2 nm. The PC Co–P coating, however, has smaller protrusions on the surface with *R*_a_ of ∼44.7 nm (Fig. 4b). The PRC Co–P coating exhibits more compact and smoother surface with the surface roughness (*R*_a_) of about 8.5 nm (Fig. 4c). The decrease of roughness can be attributed to the pulse current breaking the normal growth of cobalt crystals and disrupting larger crystals from producing smaller nuclei.^14^ Furthermore, the PRC method dissolved the unsteady phases formed in the coating by anode pulse current (especially for the protrusions of the coating surface), resulting in a smooth surface.^8^ Correspondingly, the coatings show completely different glossiness (Fig. 5). The glossiness of DC Co–P, PC Co–P and PRC Co–P coating is 5.8, 30.6 and 518, respectively. The brighter surface of amorphous coating than nanocrystalline coating is due to the higher P content and more smooth surface.^1,15^

**Fig. 6:**
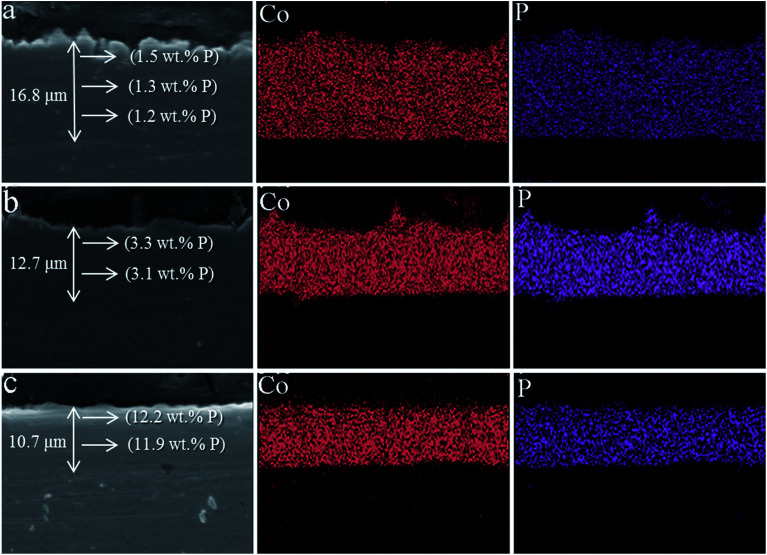
The cross-sectional SEM images of (a) DC Co–P, (b) PC Co–P and (c) PRC Co–P coatings.

In order to study the thickness of Co–P coatings and the binding of Co–P coating with Cu substrate, the cross section images of three Co–P coatings are shown in Fig. 6. It is clearly seen that the thickness of DC Co–P, PC Co–P and PRC Co–P coatings are decreasing. Compared with PC Co–P and PRC Co–P coatings, however, the surface of DC Co–P coating is very rough and has large number of cracks and voids. These results are well agreed with the surface morphology as shown Fig. 3 and 4. From the EDS analysis it could be observed that the P content of all the three Co–P coatings remained almost same throughout the coating thickness, indicating that the composition distributing of Co–P coatings was homogeneous in thickness direction from surface to interior.

### 3.2 Tribological behavior

**Fig. 7:**
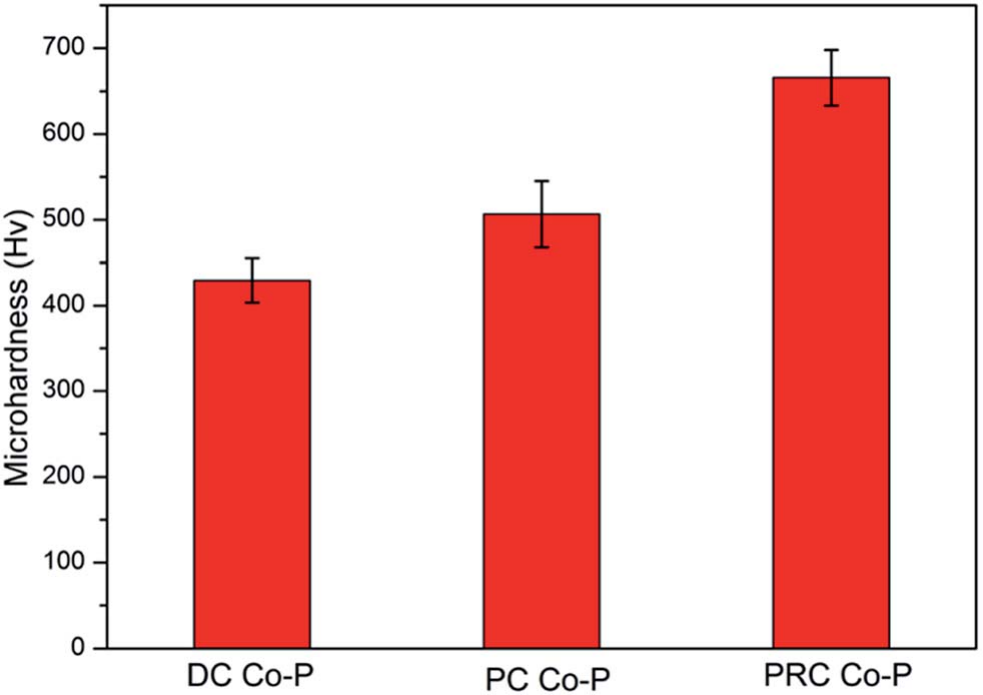
The microhardness of Co–P coatings deposited by DC, PC and PRC methods.

Microhardness is a very important performance characteristic of coating materials, especially for the tribological performances. Fig. 7 shows the microhardness of Co–P coatings deposited by DC, PC and PRC methods. The microhardness of the DC Co–P, PC Co–P and PRC Co–P coatings is 430 Hv, 506 Hv and 665 Hv, respectively. The improvement of microhardness is mainly ascribed to the refinement of grain size, the change of structure and the increase of P content.^15,16^

**Fig. 8:**
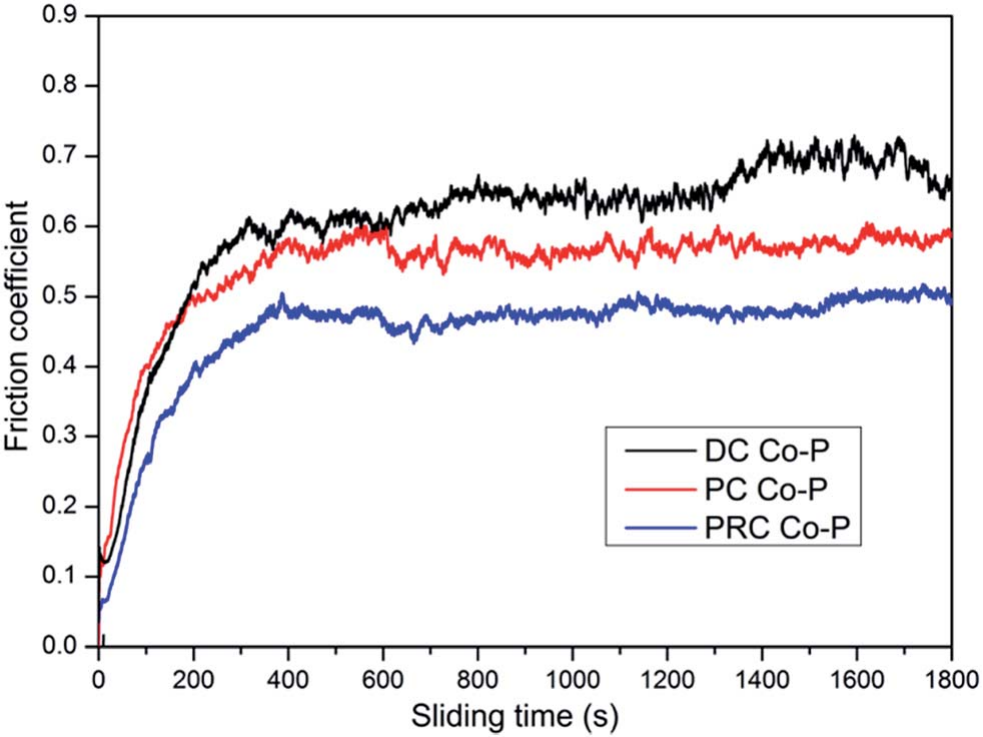
The friction coefficient of Co–P coatings deposited by DC, PC and PRC methods.

The friction coefficient of the DC Co–P, PC Co–P and PRC Co–P coatings as a function of the sliding time are shown in Fig. 8. It is noted that the DC Co–P coating exhibited higher friction coefficient (0.66) with a large fluctuation. The fluctuation of friction coefficient is due to the rough surface of the coating. The PC and PRC Co–P coating, however, shows a stable and lower friction coefficient (0.54 and 0.48, respectively). For the cobalt-based alloys, it was reported that the hcp structure exhibits lower friction coefficient and wear rate than the fcc structure.^17–19^ Therefore, the DC Co–P coating characterized by a mixed fcc/hcp structure with higher ratio of fcc shows a higher friction coefficient than the hcp structural PC Co–P coating. The PRC Co–P coating exhibits a lower friction coefficient than the PC Co–P coating, which is mainly due to its smooth surface and amorphous structure.^20^

**Fig. 9:**
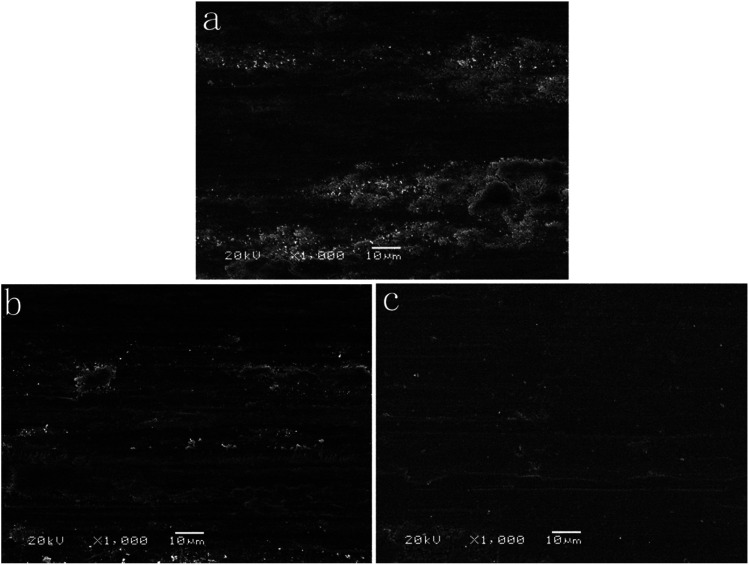
SEM micrograph of the wear tracks of (a) DC Co–P, (b) PC Co–P and (c) PRC Co–P coatings.

The wear track is an important evidence to demonstrate the tribological mechanism. As shown in Fig. 9, the DC Co–P coating (Fig. 9a) is characterized by rough worn surface, plenty of wear debris and severe plastic deformation, indicating that the wear mechanism of the DC Co–P coating is mainly adhesive wear. The worn surfaces for PC Co–P coating (Fig. 9b) are characterized by abrasive grooves along the sliding direction, less wear debris and plastic deformation. It was the classical mixed mechanism of adhesive–abrasive wear. However, the PRC Co–P coating shows smoother worn surface with a small amount of wear debris and plastic deformation, indicating that the PRC Co–P coating with hcp structure has excellent anti-adhesive wear properties.^17,21^

**Fig. 10:**
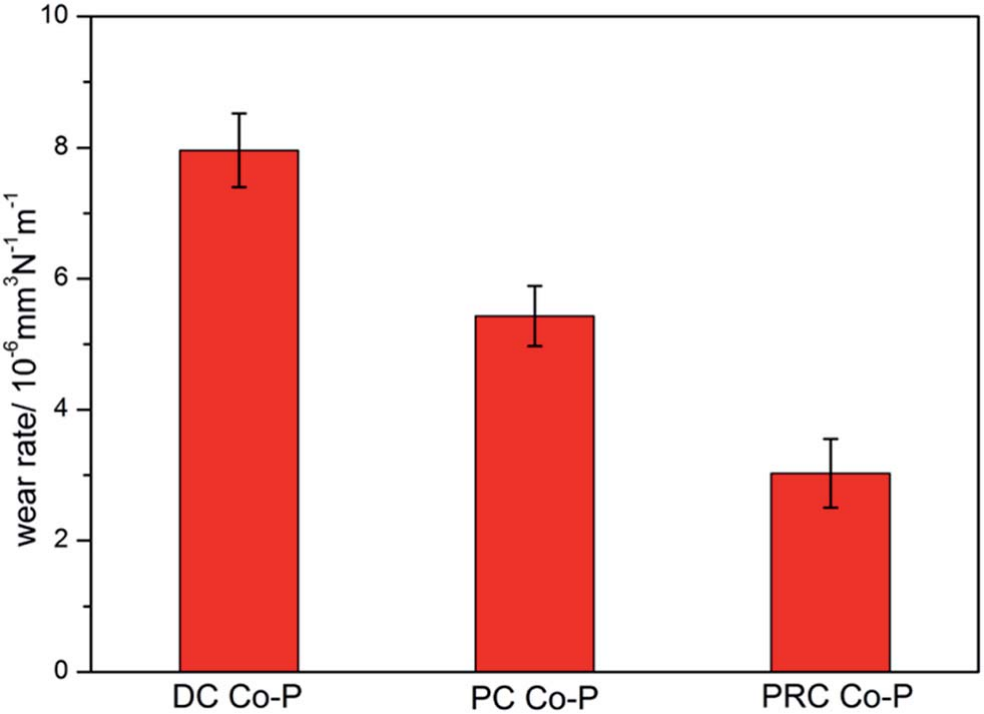
The war rate of Co–P coatings deposited by DC, PC and PRC methods.

The wear rates of the Co–P coatings deposited by DC, PC and PRC methods are shown in Fig. 10. It is noticed that the DC Co–P coating has the highest wear rate, suggesting that the wear resistance of the coating is rather weak due to the lower microhardness. Compared with the DC and PC Co–P coatings, the PRC Co–P coating exhibits the lowest wear rate, which is mainly dependent on the higher microhardness and lower friction coefficient.^20^ It reveals that the current mode can affect wear behaviors of Co–P coatings by changing crystal structure and P content of Co–P coatings.

### 3.3 Electrochemical corrosion behavior

**Fig. 11:**
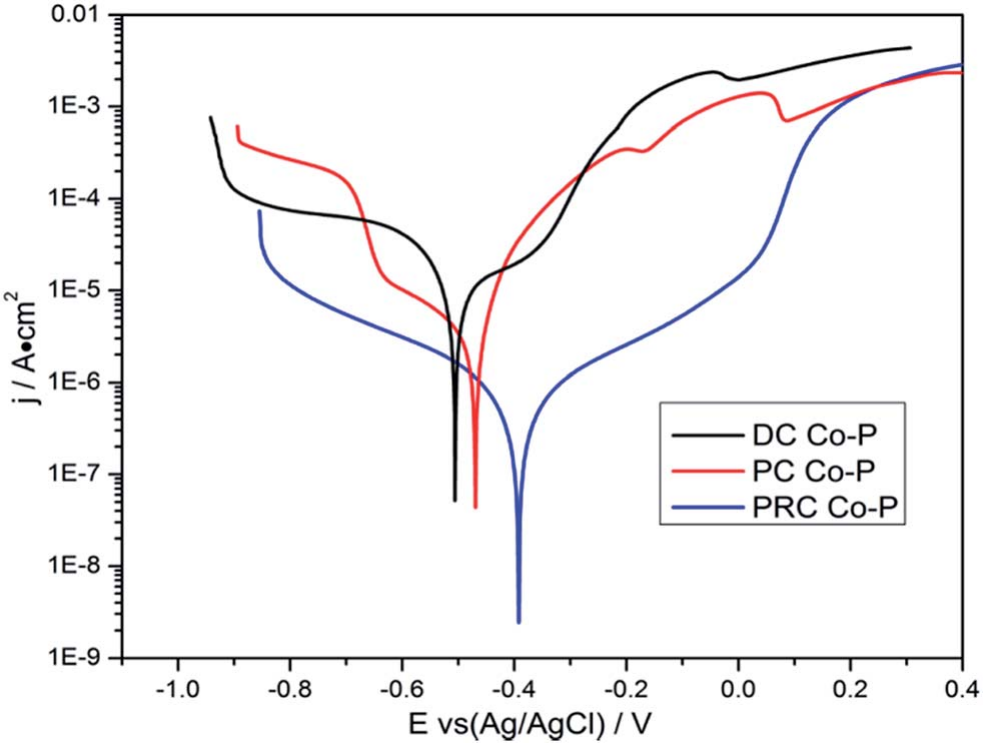
The potentiodynamic polarization curves of Co–P coatings deposited by DC, PC and PRC methods.

The potentiodynamic polarization curves of the Co–P coatings deposited by DC, PC and PRC methods in a non-deaerated 3.5% NaCl solution are shown in Fig. 11. The corrosion parameters were obtained from the intersection of cathodic and anodic Tafel curve tangents using the extrapolation method^22,23^ and the results are listed in Table 3. It is noticed that the corrosion potential (*E*_corr_) and corrosion current density (*i*_corr_) of DC Co–P coating are −0.51 V *vs.* Ag/AgCl and 9.4 × 10^−6^ A cm^−2^, respectively. The *E*_corr_ of the PC Co–P coating shifted to a slightly positive value (∼30 mV) than that of DC Co–P coating. However, the *i*_corr_ value (2.2 × 10^−6^ A cm^−2^) of the PC Co–P coating is much lower than that of the DC Co–P coating, which indicates that the PC coated alloy has a higher general corrosion resistance than that of the DC coated alloy in 3.5% NaCl solution. The lower corrosion resistance of the DC Co–P coating is mainly due to the cracks and rough spherical protrusions on the surface which are usually vulnerable to corrosion attack.^7^ It is clear that the PRC Co–P coating shows a more positive *E*_corr_ (−0.39 V *vs.* Ag/AgCl) and the *i*_corr_ value (2.0 × 10^−7^ A cm^−2^) has *ca.* twelvefold and fiftyfold reduction in comparison with the PC and DC coatings, respectively. The higher corrosion resistance of the PRC Co–P coating can be attributed to the following aspects. Firstly, the smooth and dense surface effectively inhibits the penetration of corrosive medium through the coating. Secondly, the Co–P alloy with high P content exhibits good corrosion resistance than those with low P content due to the P has tendency to form a barrier layer of hypophosphite anion when exposed to corrosive environments. The mechanism of corrosion resistance of P in the corrosive medium can be explained by a similar way as proposed for Ni–P coating.^6^ The cobalt of Co–P coatings was preferentially dissolved in the corrosive medium, leading to an enrichment of phosphorous on the surface layer. The phosphorous reacts with water to form a layer of adsorbed hypophosphite anions on the surface of coatings (eqn (1) and (2)).1
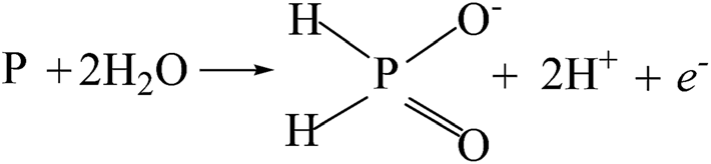
2
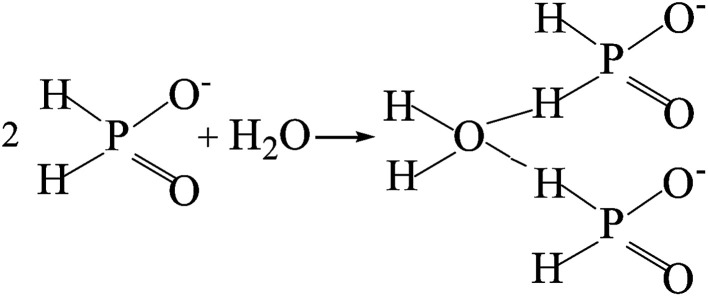


**Table 3:** Electrochemical data of Co–P coatings derived from the polarization tests in 3.5 wt% NaCl solution

Specimens	*E*_corr_ (V *vs.* Ag/AgCl)	*i*_corr_ (A cm^−2^)
DC Co–P	−0.51	9.4 × 10^−6^
PC Co–P	−0.48	2.2 × 10^−6^
PRC Co–P	−0.39	1.8 × 10^−7^

The eqn (2) shows near steady-state barrier layer of hypophosphite anion. This layer in turn prevents the water to from reaching the electrode surface, thereby preventing the dissolution of cobalt.

**Fig. 12:**
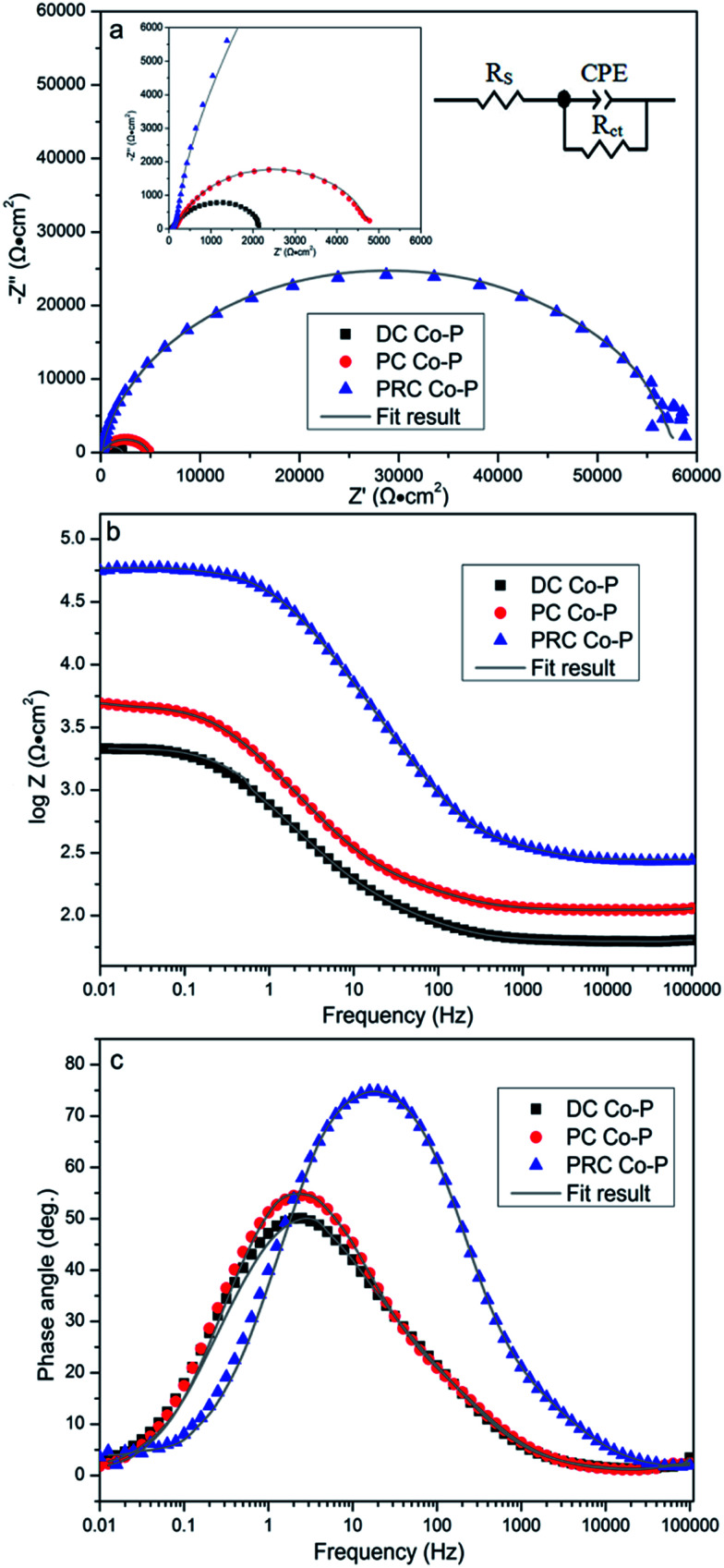
Experimental and fitting results of (a) Nyquist and (b and c) Bode plots for Co–P coatings.

The EIS tests are carried out to further investigate the corrosion resistance of the Co–P coatings. Fig. 12 shows the resulting EIS plots of various specimens in a non-deaerated 3.5 wt% NaCl solution. The Nyquist plot (Fig. 12a) exhibits depressed semicircle with a single capacitive loop in the high frequency region that indicates a charge controlled reaction. It is clear that the radius of capacitive loops decreases in sequence of PRC Co–P, PC Co–P and DC Co–P coatings, indicating that the PRC Co–P coating has a better corrosion resistance than the DC and PC coatings. It can be seen from Fig. 12a that the DC Co–P coating shows the lowest value of |*Z*|_f → 0_ (2.15 × 10^3^ Ω cm^2^). Compared with the DC coating, the value of |*Z*|_f → 0_ of the PC Co–P coating (4.93 × 10^3^ Ω cm^2^) is slightly increased, whereas the value of |*Z*|_f → 0_ of the PRC Co–P coating (5.87 × 10^4^ Ω cm^2^) is more than one order of magnitude higher than that of the PC Co–P coating.

The impedance spectra for the Nyquist plots are analyzed by fitting the experimental data to the equivalent circuit model as shown in Fig. 12a. There is only one obvious extremum in the phase angle curves (Fig. 12c), which means that there is only one time constant. A good fit with this model was obtained with an average error of about 3%. In this equivalent circuit, *R*_s_ represents the solution resistance. *R*_ct_ represents the charge transfer resistance, which is a measure of electron transfer across the surface and is inversely proportional to corrosion rate. Constant phase element (CPE) represents the capacitance of the double layer (*C*_dl_), which provides information about the polarity and the amount of charge at the surface/electrolyte interface. The fitted results of circuit elements obtained from Zview software are summarized in Table 4. From the fitted data, it can be seen that the *R*_ct_ of Co–P coating deposited by PRC method is much higher than that of the other coatings, indicating that the corrosion resistance of PRC Co–P coating is the best while the corrosion resistance of coatings deposited by DC is the worst. Furthermore, the fitted data showed that the CPE value became lower and lower in order of the DC Co–P, PC Co–P and PRC Co–P coatings, while the *n* value became higher and higher correspondingly. The lower value of CPE indicates the corrosion layers for the PRC Co–P coating is becoming less permeable and the higher *n* values suggests a homogeneous, smooth and pore-free deposits that results in a better corrosion resistance of the PRC Co–P coating.^24^ The results of corrosion resistance obtained from EIS plots are well consistent with the polarization tests.

**Table 4:** EIS fitting results of the specimens in 3.5 wt% NaCl solution

Specimens	*R*_s_ (Ω cm^2^)	CPE(Y_0_)_1_ (S cm^−2^ s^*n*^)	*n*_1_	*R*_ct_ (kΩ cm^2^)
DC Co–P	61.8	7.8 × 10^−6^	0.79	8.9
PC Co–P	63.3	3.4 × 10^−6^	0.83	17.2
PRC Co–P	67.4	8.2 × 10^−7^	0.95	63.7

## 4. Conclusions

Co–P coatings with various P contents and structures were deposited from chloride bath by DC, PC and PRC methods, respectively. The P content of the alloy coatings deposited by DC, PC and PRC, respectively, became higher and higher in order, and the coating surface became compacter and smoother correspondingly. The low P content coatings deposited by DC and PC methods are crystalline with fcc and hcp structure, respectively, whereas the high P content coating obtained from PRC method is amorphous. The Co–P coating deposited by PRC method exhibits much higher microhardness, wear resistance and corrosion resistance than that by DC and PC methods. It reveals that the current modes can affect the performances of Co–P coatings by changing composition, morphology, crystal structure and grain size of Co–P coatings.

## Conflicts of interest

There are no conflicts to declare.

## Supplementary Material
